# Novel Dermal Delivery Cargos of Clobetasol Propionate: An Update

**DOI:** 10.3390/pharmaceutics14020383

**Published:** 2022-02-09

**Authors:** Anroop B. Nair, Sunil Kumar, Pooja Dalal, Chahat Nagpal, Sweta Dalal, Rekha Rao, Nagaraja Sreeharsha, Shery Jacob

**Affiliations:** 1 Department of Pharmaceutical Sciences, College of Clinical Pharmacy, King Faisal University, Hofuf 31982, Al-Ahsa, Saudi Arabia; sharsha@kfu.edu.sa; 2 Department of Pharmaceutical Sciences, Guru Jambheshwar University of Science and Technology, Hisar 125001, India; sunilkundu450@gmail.com (S.K.); lamba808@gmail.com (P.D.); chahatnagpal0001@gmail.com (C.N.); swetadalal143@gmail.com (S.D.); 3 Department of Pharmaceutics, Vidya Siri College of Pharmacy, Off Sarjapura Road, Bangalore 560035, India; 4 Department of Pharmaceutical Sciences, College of Pharmacy, Gulf Medical University, Ajman 4184, United Arab Emirates; sheryjacob6876@gmail.com

**Keywords:** dermal delivery, stability concerns, safety concerns, nanoformulations, in vivo studies, cell line studies

## Abstract

Dermal disorders such as psoriasis and eczema are associated with modifications in the chemical and molecular composition of the skin. Clobetasol propionate (CP), a superpotent topical glucocorticoid, is widely used for the therapeutic management of various skin conditions, owing to its strong anti-inflammatory, antipruritic, vasoconstrictive, and antiproliferative activities. Safety studies demonstrated that CP is safer for a shorter period, however, with prolonged application, it shows secondary side effects such as photosensitivity, Cushing-like syndrome, allergic contact dermatitis, osteonecrosis, hypopigmentation, steroid acne, and skin atrophy. Therefore, the US FDA (United States Food and Drug Administration) has restricted the usage of CP to not more than 15 days. Research scientists addressed its several formulations and drug delivery issues, such as low water solubility, less stability, photodegradation, and poor absorption, by incorporating them into novel nanobased delivery platforms. With the utilization of these technologies, these drawbacks of CP have been resolved to a large extent to reestablish this moiety. This article explores the physicochemical properties and mechanism of action of CP. Additionally, an attempt has been made to discover and highlight the possible features of the novel nanosystems, including nanoemulsions, nanosponges, solid lipid nanoparticles, nanostructured lipid carriers, and nanogels, reported for CP. The stability and safety concerns of CP, along with its commercial status, are also discussed.

## 1. Introduction

Glucocorticoids are very effective moieties extensively employed in the therapeutic management of inflammatory and pruritic presentations of various dermatological conditions. Nevertheless, serious side effects are frequently noticed with their continuous use. For topical application, steroidal formulations are divided into four groups, as per their therapeutic performance: mild, moderate, potent, and super potent [1]. Among them, clobetasol propionate (CP), a synthetic compound, belongs to the “strongest” group i.e., most potent topical glucocorticoids and is one of the widely prescribed FDA-approved prescription medicines available in the market [2]. Conventional formulations of CP available for dermatological applications include foams, creams, gels, ointments, lotions, shampoos, and sprays [3]. Novel formulations of CP, namely, Cormax^®^, Clobex^®^, Dermovate^®^, and Temovate^®^, are also commercially available for the relief of the inflammatory and pruritic manifestations of corticosteroid-responsive dermatoses [4].

CP promotes induction of phospholipase A2 suppresser proteins, thus controlling the release of the inflammatory precursor, arachidonic acid, from membrane phospholipids via the above-mentioned enzyme [5]. This topical synthetic corticosteroid possesses vasoconstriction potential over 1800-fold higher than hydrocortisone. However, associated adverse effects are comparatively higher than related compounds [6]. The reported adverse side effects include skin atrophy, hypopigmentation around the application site, steroid acne, allergic contact dermatitis, Cushing-like syndrome, adrenal hypophysial axis suppression, osteonecrosis, dermal striae, telangiectasia, tachyphylaxis pruritus, and folliculitis [7,8].

CP has been explored against numerous skin diseases such as atopic dermatitis, vitiligo, psoriasis, eczema, and autoimmune cell-mediated disorders [1]. Taking into account its massive curative perspective, CP has been approved by the US FDA for the management of inflammatory skin disorders [9,10]. Not only this, CP has become one of the most popular corticosteroids used as a first-line approach for diverse skin diseases [1]. Hence, CP application has been drastically enhanced over the past few decades.

Although potent, CP is a pharmaceutically challenging molecule due to poor water solubility and susceptibility to photodegradation. Even though it shows potential clinical value, it promotes secondary unfavorable effects because of systemic absorption after topical application. Therefore, the FDA restricts continuous utilization of this drug to a maximum period of 2 weeks [7]. For prevention and management of adverse effects, nowadays, researchers have been continuously paying attention to the development of a variety of drug delivery payloads. Such novel cargos are currently in progress to enhance the curative benefits and safety of this potent therapeutic agent. Additionally, these are intended to transport CP in a modulated way within the different skin layers. Other key benefits of novel cargos are their abilities to provide a more effective concentration of the active therapeutic agent at the target site, to decrease the severity of the undesirable effects, to minimize dermal clearance, to increase the diffusion rate of the drug through epithelial layers, and to possibly improve patient compliance. In general, physical and chemical approaches are widely used to enhance the topical and transdermal delivery of various therapeutic moieties [11,12,13]. However, for effective topical delivery, a prolonged contact time of the drug on the skin surface or within the epidermis, while preventing its penetration in systemic circulation, is needed [4]. Novel cargos possess a great efficacy to augment the potential of active moieties by enhancing their therapeutic efficacy and minimizing their toxicity. Furthermore, this reduces the overall dose and consumption of active moiety [14,15].

**Figure 1:**
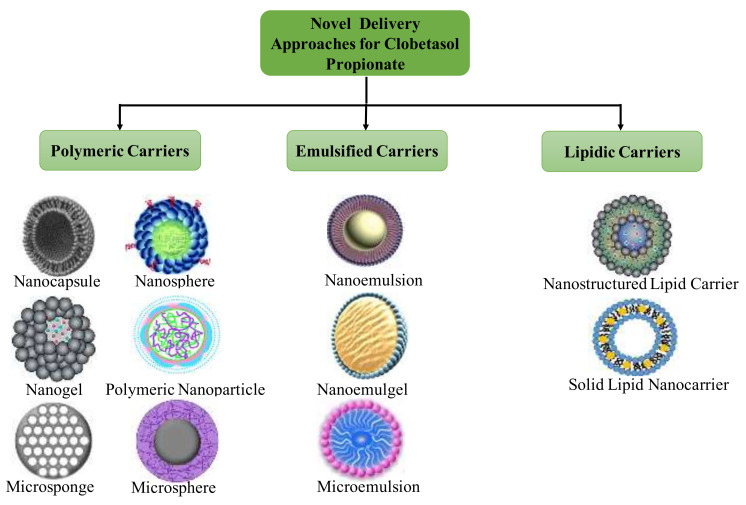
Novel cargos for clobetasol propionate delivery.

The profound potential of CP has attracted our great interest to review pharmaceutical research performed on this activity in the last few years. Unexpectedly, no review article is presented on CP, which is a backbone for the management of diverse skin ailments, for the last decade. This article is a brief description of the blueprint of novel colloidal drug delivery carriers incorporating CP, namely microspheres, polymeric nanoparticles, nanostructured lipid carriers (NLCs), solid lipid nanoparticles (SLNs), nanoemulsions, nanocapsules, and nanogels (Figure 1). Recently, our group has investigated the targeting ability of this moiety by formulating as microsponges [4], nanosponges, and nanosponge-loaded hydrogels [14]. Furthermore, the focus of this review is the assessment of obstacles and the success of these novel carrier systems for facilitating effective CP delivery and its dermal targeting. This appraisal also represents an effort to furnish an outline of in vitro and ex vivo research performed for CP. Additionally, a brief account of stability and safety concerns for CP has also been provided. It is an effort to give a bird’s- eye view to the drug professionals about signs of progress in this area. This review article will surely provide fresh perspectives for scientists for exploring this molecule.

## 2. Physiochemical Properties and Metabolism of Clobetasol Propionate

**Figure 2:**
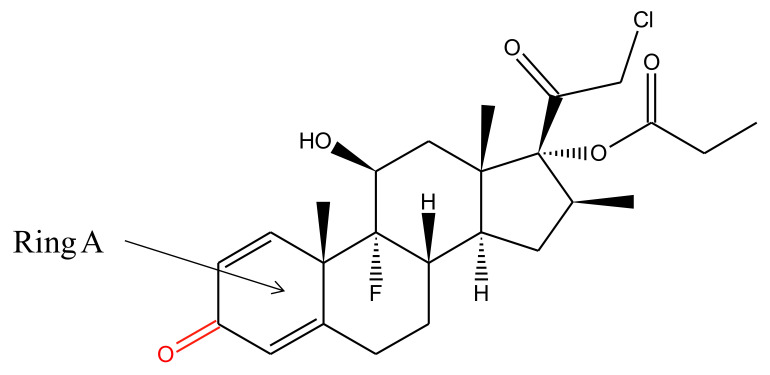
Chemical structure of clobetasol propionate.

Clobetasol-17-propionate is an odorless, white to cream-colored crystalline powder, having solubility 2 μg/mL in water [16]. The chemical structure of this moiety is shown in Figure 2. It is an analogue of prednisolone with a molecular weight of 466.97 g/mol, a melting point of 196.25 °C, and a log *p* value of 2.98 [17]. Its chemical name is [(8*S*,9*R*,10*S*,11*S*,13*S*,14*S*,16*S*,17*R*)-17-(2-chloroacetyl)-9-fluoro-11-hydroxy-10,13,16-trimethyl-3-oxo-6,7,8,11,12,14,15,16-octahydrocyclopenta[a] phenanthren-17-yl] propanoate, with the empirical formula C_25_H_32_ClFO_5_.

CP has characteristic UV absorption at λ_max_ 237 nm (in methanol). It is a very attention-grabbing moiety from the photochemical point of view because it has two spatially divided chromophores, i.e., carbonyl group at C-20 and cyclohexadiene moiety in ring A [18].

Hepatic microsomal enzymes metabolize corticosteroids through the reduction of 20-keto to 20-hydroxy form, the hydroxylation of 3-keto group, and the reduction of 4, 5 double bonds. Furthermore, the metabolites are conjugated with either sulfate or glucuronic acid and excreted in the urine. After dermal administration, there is very little systemic absorption observed for CP [17].

## 3. Mechanism of Action of Clobetasol Propionate

**Figure 3:**
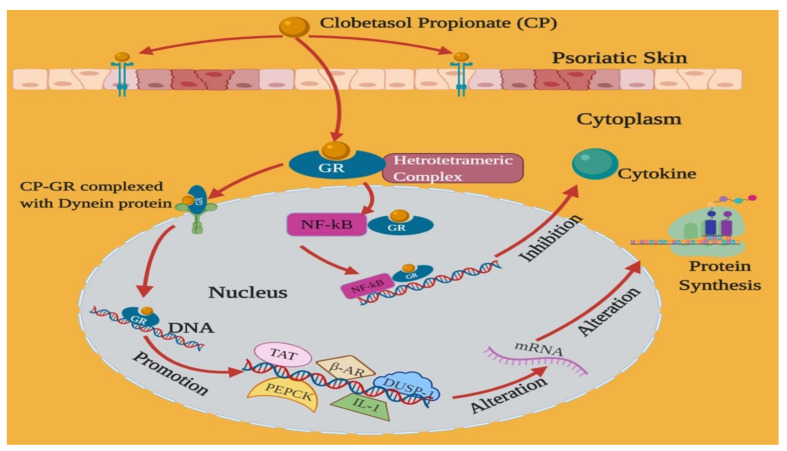
Mechanism of action of clobetasol propionate after topical application. GR—Glucocorticoid Receptor; TAT—tyrosine aminotransferase; PEPCK—phosphoenolpyruvate carboxykinase; β-AR—beta-adrenergic receptor; DUSP—dual-specificity protein phosphate; NF-κb—nuclear factor kappa-light-chain-enhancer;IL-1—interleukin 1; mRNA—messenger RNA.

CP is usually prescribed for the treatment and management of eczema, psoriasis, contact dermatitis, dry hyperkeratotic dermatoses, atopic dermatitis, discoid lumps erythema, lichen planus, and granulomatous disorders [10,17]. At the cellular level, corticosteroids proceed in two diverse pathways (nongenomic and genomic). In the genomic pathway, cortisol activates the glucocorticoid receptors (GR) and consequently results in receptor homodimerization as well as binding to GREs (glucocorticoid-responsive elements). The mechanism of action of CP is depicted in Figure 3.

The nongenomic path is accountable for the immediate therapeutic effect of glucocorticoids. In this pathway, second messengers and membrane-bound receptors are particularly taken into consideration. This course acts via modulating the activation levels and the response of the target cells (monocytes, platelets, and T cells) and does not necessitate de novo protein production [1].

Glucocorticoids initially bind to the steroid-binding site of intercellular GR, followed by translocation to the nucleus, finally altering the gene transcription. In an unbound (steroid-free) state, intracellular GR is bound to immunophilin and to heat-shock protein 90 (Hsp90) (stabilizing proteins). The steroid-free (unbound) state of the receptor is not able to affect the gene transcription. Steroid binding starts a conformational alteration that leads to an exchange of chaperone proteins. These proteins allow attaching the steroid-GR complex with the dynein protein trafficking pathway, thereby translocating the steroid-GR complex from the cytoplasm to the nucleus. The promotion of anti-inflammatory functions (phosphoenol pyruvate carboxykinase (PEPCK), dual-specificity protein phosphatase 1 (DUSP-1), tyrosine aminotransferase (TAT), β-adrenergic receptor, IL-10, and IL-1-receptor antagonist) occurs after transcription of genes via GR dimerization, palindromic promoter sequence binding, and GREs. The binding of the glucocorticoid-GR dimer either stimulates or represses the transcription of susceptible genes, ensuring the variations in mRNA synthesis, followed by alterations in the synthesis of protein [19].

In addition to directly regulating gene transcription, corticosteroids are capable of managing transcription indirectly via blockage of other transcription factors. Particularly, these moieties have been revealed to raise the inhibitory nuclear factor-κBa (IκBa) at cellular levels through stimulation of IkBa gene expression. In turn, IkBa protein prevents transcription via binding with another transcription regulator, NF-kB (nuclear factor-kB), to avoid translocation to the nucleus. Corticosteroids possibly influence the gene transcription, which does not have glucocorticoid-susceptible receptors [20].

Corticosteroids have also been established to decrease the proliferation of T lymphocytes. The arachidonic acid metabolic products formation can be inhibited by CP. However, the antiproliferative action of these actives has not been clear but may comprise both suppressions of cytokine effects and blockades of cytokine expression [19]. Declined levels of leukotrienes, prostaglandins, and arachidonic acid metabolic products have been reported from the serum of diseased persons who were taking glucocorticoids for various inflammatory skin disorders [7].

## 4. Novel Formulations Reported for Clobetasol Propionate

The topical use of novel cargos is a topic of frequent discussion and exploration, as these cargos possess numerous advantages over conventional preparations as discussed in the introduction. The research on the definite mode of action of moieties loaded in these cargos should be carried out for their clinical utility. Nevertheless, among these, the safety of potential bio-persistent nanomaterials and their by-products within the dermis annexes is a matter of concern. The novel formulations discussed in this segment (Table 1) have been principally explored to improve the therapeutic efficacy of CP and to overcome its associated challenges. Some formulations are also aimed for solubility enhancement and prolonged release of this moiety.

### 4.1. Nanoemulsions

Nanoemulsions are kinetically stable but thermodynamically unstable and optically clear dispersions of multicomponent fluids and are generally constituted as an aqueous phase, an oily phase, a primary surfactant as an emulsifying agent, a cosurfactant an alkanol of intermediate chain length, and an electrolyte [41]. Nanoemulsions are milky, translucent, or transparent depending on the size of droplets. Based on the chemical nature and proportion of components employed, these are categorized into two types, oil in water (*o*/*w*) and water in oil (*w*/*o*). Low or high-energy emulsification techniques are employed for their preparations. The small droplet size of these preparations (commonly 100–500 nm of the size range) makes them resistant to creaming, flocculation, and physical destabilization [42,43,44]. The key benefits include an increase in dermal retention and prolonged duration, reduced drug-protein binding, improved drug permeation, sustained drug release, reduced systemic adverse effects, and ease of use for the inclusion of both hydrophilic and hydrophobic drugs [45,46,47].

Alam et al. fabricated oil in water (*o*/*w*) nanoemulsions comprising Tween^®^ 20 (Polysorbate 20), eucalyptus oil, ethanol, and distilled water using an aqueous phase titration technique for improvement of CP dermal delivery. To investigate nanoemulsion region, pseudo ternary phase diagrams were made for each S_mix_ ratio (combination of surfactant and cosurfactant). Finally, S_mix_ (1:2) was chosen for the fabrication of the nanoemulsions. Optimized nanoemulsions were fabricated by dissolving CP (0.05% *w*/*v*) in eucalyptus oil (10% *v*/*v*) and consequently, 35% *v*/*v* mixture of Tween^®^-20 and ethyl alcohol (1:2 *v*/*v*) was added slowly in the oil phase. The prepared CP-loaded nanoemulsions were assayed for in vivo anti-inflammatory potential, irritation studies, and NTPDase (Nucleoside triphosphate phosphohydrolase) activity in lymphocytes. Contact dermatitis was induced by 5% nickel sulfate in solid Vaseline. The dose of application for nanoemulsion and the marketed formulation was equal to 1 mg of clobetasol propionate. A test group and a positive control were applied daily with 0.5 mL of CP-loaded nanoemulsion and marketed cream for 5 days (on the 1st, 3rd, and 5th day). A remarkable enhancement (*p* < 0.05) in NTPDase potential was observed in lymphocytes in the CP-loaded nanoemulsion treated group compared to all other treatment groups. The nanoformulation was found to possess enhanced anti-inflammatory action and showed no sign of irritation (despite the presence of high surfactant), ascertaining its safety and efficacy [21].

The same research group also investigated nanoemulsions for dermal delivery of CP by employing algal oil (having omega-3 fatty acids). Owing to the poor skin permeability of CP, drug-loaded algal oil nanoemulsion was explored for anti-inflammatory activity in the management of dermatitis. In this study, nanoemulsions were fabricated employing the aqueous phase titration technique, using algal oil, PEG-200 (Polyethylene glycol-200), Tween^®^ 20 (Polysorbate 20), and water. Furthermore, the fabricated cargos were evaluated for their in vivo permeation potential, in vivo irritation, and anti-inflammatory activity in skin dermatitis. The selected nanoemulsion was embedded into hydrogel employing Carbopol^®^ 971 (viscosity: 97.57 ± 0.04 PaS). The fabricated nanoemulsions (having 0.05% *w*/*w*) demonstrated average droplet size (120 ± 1.95 nm), PdI (0.325 ± 0.02), and satisfactory zeta potential (−37.01 mV). The anti-inflammatory potential of CP-loaded nanoemulsion hydrogel (0.05% *w*/*w* of CP) was compared with placebo nanoemulsion hydrogel. In vivo anti-inflammatory potential ascertained 41.04% and 84.55% inflammation inhibition for placebo and drug-loaded nanoemulsion hydrogel, respectively. The skin permeation assessed by histopathology and thermal analysis showed alterations in skin strata (epidermal membrane). The skin irritation score for CP-loaded nanoemulsion gel (1.66 ± 0.81) and placebo nanoemulsion gel (0.83 ± 0.75) based on a 14-day test concluded the safe nature of optimized nanoemulsion for dermal delivery. The nanoemulsions presented a higher skin irritation score owing to the higher content of surfactant than placebo nanoemulsions and the inflammatory nature of CP. Contact dermatitis was induced by 5% nickel sulfate in solid Vaseline. After tricotomization, all animals in treatment groups (except the group treated with only solid vaseline) received sensitization with nickel sulfate in the abdomen. The dose of application for nanoemulsion and the marketed formulation was equal to 1 μg of CP. All treatment formulations were applied uniformly throughout the ear tissue with massage for better penetration of CP. After the complete treatment procedure, the animals were euthanized, and the blood was collected by cardiac puncture to examine the NTPDase potential. For NTPDase activity of lymphocytes, mononuclear leukocytes were isolated from rat blood collected with EDTA and separated using Ficoll-Hypaque density gradients. After the isolation of mononuclear cells, NTPDase potential was examined by colorimetry. In vivo contact dermatitis study revealed that CP-loaded nanoemulsion gel potentially enhanced NTPDase activity [22].

For the management of psoriasis, Kaur and her research group designed nanoemulsions for codelivery of CP and calcipotriol employing surfactant/co-surfactant and oil-based on their emulsification and solubility potential, respectively. Capmul^®^ MCM C8 EP (Glyceryl caprylate), Cremophor^®^ RH 40 (PEG-40 Hydrogenated Castor Oil), and Labrafil^®^ 1944 CS (hexadecanoic acid) were employed in a 5:3:2 ratio, respectively for nanoemulsion fabrication. Furthermore, the formulations were embedded into Carbopol^®^ 980 to obtain the final CP concentration of 0.05% *w*/*w* and calcipotriol concentration of 0.005% *w*/*w*. The cell uptake studies performed using HaCaT cell lines presented higher drug uptake from formulation (equivalent to 1 μg/mL of free coumarin-6) than from free coumarin 6. The formulations (about 200 mg having 0.05% *w*/*w* of CP and 0.005% *w*/*w* of calcipotriol) were applied on dorsal hairless skin with the help of clean cotton for 2 h. TEWL (trans epidermal water loss) was checked by employing a vapometer before cell treatment, and then daily (4 days) during treatment. Skin irritation was evaluated based on erythema or redness by visual observations. Capsaicin presented maximum irritation potential and high values of % enhancement in TEWL. Free CP and calcipotriol (CP-CT) embedded gel showed higher irritation potential than nanoemulsion gel, and hence was better tolerated. The order of skin irritation score for different samples was presented as capsaicin > free CP-CT gel > nanoemulsion gel. Higher drug penetration in rat skin was found responsible for irritation. Moreover, in the case of drug-loaded nanoemulsion gel, there was minimum direct contact of the drug with the skin. Furthermore, the nanoemulsion gel enhanced CP concentration locally, resulting in a decrease in its systemic adverse effects in comparison to commercial dosage forms. The higher antipsoriatic potential was also exhibited by nanoemulsion gel, when compared to the free drug and the marketed commercial dosage form with negligible skin irritation [23].

### 4.2. Chitin Nanogel

Nanogels, hydrogel, or self-assembled nanoparticles, have received wide attention as one of the most attractive nanoparticulate drug delivery systems because they combine unique hydrogel characteristics with submicron particle size [48]. The drug molecule can be either conjugated to the surface of the nanoparticles or embedded within the matrix. The surface charge, hydrophobicity, and particle size can be modified for both active and passive targeting. In addition, controlled or sustained release, ability to reach smaller blood vessels permeation to tissue via the paracellular or transcellular pathway, and feasibility for administration through various routes are other benefits of this novel colloidal carrier system [49].

CP-loaded chitin nanogel was fabricated using chitin and calcium chloride by Panonnummal et al. in 2017. The fabricated nanogel was characterized for consistency, stability, and particle size (132.0 ± 14 with a polydispersity index (PdI; 0.436), and zeta potential (+30 mV). The MTT (3-(4,5-Dimethylthiazol-2-yl)-2,5-Diphenyltetrazolium bromide) assay was conducted using HaCaT and THP1 cell lines to better understand the toxicity of CP-loaded chitin nanogel. The HaCaT cells were analyzed under a fluorescent microscope for the investigation of nanogel uptake. The nanogel displayed 70% of LOX (lipoxygenase) inhibition and 65% of COX (cyclooxygenase) inhibition expressed in THP1 cell lines. The transdermal flux in epidermal layers and stratum corneum was found to be enhanced on treatment with CP chitin nanogel, affecting the control drug solution. An in vivo imiquimod induced psoriasis mouse model revealed the potential benefits of CP-loaded chitin nanogel in dermal delivery for psoriasis management [7].

### 4.3. Solid Lipid Nanoparticles

SLNs are nanosized (10–1000 nm) particles fabricated from solid lipids dispersed in aqueous media comprising surfactants as stabilizers. The incorporation of physiologically compatible lipids minimizes toxicity and irritation potential frequently encountered in other nanoparticulate systems. Other advantages are sustained and controlled drug release, excellent physical stability, resistance to degradation, in vivo acceptability, and adaptability to various drug delivery systems [45,50].

Hu et al. fabricated CP-loaded SLNs using the solvent diffusion technique. The fabricated formulations were evaluated for particle size, zeta potential, PdI, and encapsulation efficacy. The recovery of SLNs was markedly enhanced using the acidic aqueous medium in comparison with commonly used aqueous (pH 5.73) medium possessing equal concentration (1%) of polyvinyl alcohol. After an initial burst release (in the first 3 h), approximately 6% drug release was observed daily (for four days), demonstrating the suitability of SLNs for prolonged application [51].

Kalariya and coworkers synthesized CP-loaded SLNs via the high-pressure homogenization method and evaluated them for average particle size, topography, and encapsulation potential. Selected CP-loaded SLNs were observed smooth and spherical, with mean particle size 177 nm and drug encapsulation of 92.5%. Furthermore, these were incorporated into a cream base. Human cadaver skin was employed for skin uptake studies and drug permeation experiments using CP cream and CP SLN cream. The results demonstrated low flux value and high dermal uptake of the drug from CP-loaded SLN cream concerning commercial CP cream. Patients (16) with chronic eczema were then used for a controlled double-blind clinical trial. CP-loaded SLN application displayed significant improvement in the manifestation of chronic eczema (1.9 times inflammation and 1.2 times itching) as compared to marketed cream. The results of this work ascertained the efficacy of prepared nanoformulations for the management of eczema [52].

In 2019 Reddy and a research group fabricated CP-loaded SLN using the emulsification-homogenization technique for achieving high CP topical delivery as well as to investigate the influences of different independent variables including surfactant, homogenization time, and lipid:drug ratio on particulate features of the nanoformulation. The optimized nanoformulation displayed 133.3 ± 3.66 nm size of particles, 0.179 ± 0.081 of PdI, −36.2 ± 0.11 mV of zeta potential, and 78.1 ± 1.11% of entrapment efficiency. Investigation of skin permeation of formulated CP-SLN suspension presented the sustained release of CP molecule up to 24 h. Maximum drug deposition (48.22 μg/mL) was attained after formulating CP into SLN in comparison to the drug deposition (19.12 μg/mL) of pure CP. SLN has been observed as a potential colloidal particulate carrier system owing to its sustained drug release for a longer time and enhanced skin permeation for CP molecules [25].

### 4.4. Nanostructured Lipid Carriers

Solid matrices of NLCs enhance the stability and safety, provide controlled drug release, and have the capacity to incorporate both hydrophilic and hydrophobic molecules, and can be delivered through various routes. The major difference between NLCs and SLNs is the type of lipids used; fluid lipids are used in NLCs; solid lipids are used in SLNs [45,50].

NLC represents one of the efficacious lipidic carrier systems for dermal delivery of actives. Silva et al. fabricated this system for CP using lecithin, taurodeoxycholate, stearic acid, and oleic acid via a microemulsion technique for topical administration. Besides characterization of the prepared NLCs, these were evaluated for particle size distribution (average particle size: 157.9 ± 19.7 nm; PdI: 0.19 ± 0.01), zeta potential (−53.9 ± 1.1 mV) and encapsulation efficiency (98.6 ± 0.4%). In vitro penetration experiments were carried out via a porcine ear in a Franz-type diffusion cell, and samples were analyzed using HPLC. The findings from the above experiments revealed higher CP accumulation in stratum corneum with NLCs as compared to an aqueous solution of CP. Hence, the prepared NLCs in this investigation demonstrated significant CP targeting in the stratum corneum [26].

In another study, CP-loaded NLC gel was developed for the management of eczema. Prepared NLCs were examined for their morphology, particle size, surface charge, encapsulation efficacy, and in vitro drug release for selecting the optimized formulation. The chosen NLCs displayed particle size (137.9 nm), zeta potential (−20.5 mV), PdI (0.224), entrapment efficiency (78.5% ± 0.03), and cumulative drug release (85.42% up to 24 h). The selected NLCs were further embedded into a gel and characterized for rheology, drug content, release kinetics, and ex vivo permeation experiments. The in vitro release kinetics studies revealed the Korsmeyer–Peppas model as the best fit model, ascertaining non-Fickian diffusion phenomenon (diffusion and swelling controlled release) for the selected formulation. The significant improvement in permeability coefficient, steady-state flux, and enhancement ratio demonstrated the promising potential of NLC-loaded gel as compared to the marketed dosage form. Additionally, the in vivo anti-inflammatory effect of NLC based gel employing the paw edema mouse model revealed the fast and prolonged onset of action of the prepared nanoformulation [27].

In the same year, to investigate epidermal targeting, Silva and his research team fabricated CP-loaded NLCs using the microemulsion method. NLCs were further coated with chitosan. All the prepared batches of NLCs were analyzed for particle size, morphology, and in vitro release studies. The permeation studies were also performed in two ways to evaluate epidermal targeting. First, the SC was detached from the lasting skin, followed by separation of the whole epidermis from the dermis part of the skin. Quantification of CP from chitosan-coated (20.26 ± 2.77 μg/cm^2^) and uncoated NLC formulations (17.85 ± 0.49 μg/cm^2^) displayed 80 times more of the drug in the epidermis compared to the marketed product (0.22 ± 0.02 μg/cm^2^). Hence, this investigation ascertained the epidermal layer targeting potential of the NLC formulation [28].

### 4.5. Nanocapsules

Nanocapsules are colloidal carrier systems in which the drug is confined to a cavity surrounded by a characteristic polymeric membrane [45]. In 2010 Fontana et al. fabricated CP nanocapsules via an interfacial deposition method using preformed polymers and characterized them appropriately. The polymers poly(lactic-co-glycolic acid) [PLGA] and poly(DL-lactide) [PLA] were employed for nanocapsule fabrication. The nanocapsules displayed high entrapment efficiency (near 100%), uniform particle size (180–200 nm), satisfactory PdI (<0.20), acidic pH (3.38 ± 0.03 to 3.83 ± 0.11), and negative zeta potential (−9.37 ± 2.28 to −7.46 ± 0.26). In vitro release studies conducted via the dialysis bag method presented biphasic drug release from all nanoformulations without showing any effect of hydrophilic polymers. The PLGA nanocapsules showed low stability due to drug leakage during their storage (3 months). The photostability studies performed under UVA radiations revealed improvement in CP photostability, owing to its encapsulation in PLA and PLGA nanocapsules. The PLA and PLGA nanocapsules were considered as a profound delivery system for photosensitive moieties [29].

In the following year, Fontana and coworkers developed CP-loaded nanocapsules/nanoemulsions for the management of contact dermatitis. The prepared nanomedicines were embedded into hydrogels for evaluating the effect of polymers. The hydrogels were examined for pH, CP content, and rheology behavior. In vitro release studies showed the best results for nanocapsule hydrogels when compared to nanoemulsion hydrogels and plain drug-loaded hydrogels. For in vivo evaluation, contact dermatitis was induced by the application of 5% nickel sulfate in vaseline (5 times used with 72 h intervals) in each ear after tricotomization. The fabricated formulations were uniformly applied daily over each ear with massage for 5 days to attain improved penetration of CP. After euthanization, blood samples were collected to determine in vivo NTPDase activity in lymphocytes. The results indicated the promising efficacy of fabricated nanosystems against the propagation of contact dermatitis. Overall, the findings from this study ascertained the profound potential of CP-loaded nanocapsule hydrogels for controlled delivery and better therapeutic efficacy [24].

De Andrade and his research team formulated CP-loaded lipid core nanocapsule (LCNC) hydrogel to investigate its in vitro skin permeation/penetration. As per the results of the permeation study, CP was not permeated in the receptor medium during the experiment (24 h) regardless of the formulation type, whereas in the case of skin penetrations examinations, penetration of CP to SC, epidermis, and dermis was found 5.8, 6.9 and 3.7 times lower for the CP nanoformulation hydrogel with respect to the CP hydrogel after 24 h. Although nanoencapsulation affected the relative CP amount in skin annexes, lipid core nanocapsule hydrogel retarded the amount of CP released 5.8, 6.9, and 3.7 times into the stratum corneum, epidermis, and dermis, respectively. This may be owing to the controlled drug permeation into the skin annexes. Furthermore, LCNC decreased the viability of systemic absorption. The findings reinforced that this nanocarrier system may act as an effective dermal nanomedicine for the management of skin disorders [40].

### 4.6. Nanoparticles

The main advantages of polymeric nanoparticles are site-specific targeting, control or sustained drug release, amenability to various routes of administration, maximum drug utilization, ability to cross the blood-brain barrier and high bioavailability, improved dissolution for low aqueous soluble drugs, and prevention of biological drug degradation [45,53,54].

Fontana and his research group fabricated different polymeric nanoparticles (nanospheres, nanocapsules) and nanoemulsions for CP. The physicochemical characterization parameters of fabricated nanoformulations were monitored via encapsulation efficacy, particle size, PdI, pH, and zeta potential for 9 months. During the storage period, in the case of nanocapsules, these parameters were found unchanged except for pH, while the increase in particle size and PdI were observed for nanospheres and nanoemulsions. In vitro release experiments displayed controlled drug release in the order: nanocapsules > nanospheres > nanoemulsions. The photostability studies carried out under UVA radiations advocated the protection of drugs (nanocapsules > nanoemulsions > nanospheres) from degradation after their encapsulation [55].

In another study, Mathes and his research group formulated CP-loaded nanocapsules, nanospheres, and lipid core nanocapsules to improve the targeting potential in the hair follicles. All three formulations were found to follow the desired sustained release pattern. The fabricated nanoformulations were further embedded in the hydrogel. The differential stripping method was employed for mechanistic investigation via follicular uptake study (labeled partially with Rhodamine B), which was found higher in nanocapsules in comparison to other prepared nanoformulations. The quantitative measurement of CP from prepared formulations (lipid-core nanocapsules, nanocapsules, and nanospheres) and Rhodamine B labeled nanocarriers revealed equivalent uptake, while a negligible quantity of the drug was observed in hair follicles in the case of free drug dispersion or hydrogel. In addition to the effects of nanoparticle types and type of dosage form on the degree of follicular permeation, the influence of massage (applied pressure ~2N; 3 min) after administration of the dosage form was noticed. Nevertheless, no potential variance was found between both dosage forms (dispersion and hydrogel) without massage. However, a higher CP uptake into hair follicles was shown after massage. Massage helped in moving the hairs as a gear pump and carried the particles into the hair follicle. CP-loaded nanocapsules displayed higher follicular recovery when compared to nanospheres and lipid-core nanocapsules with the aid of massage. A more flexible supramolecular structure of nanocapsules (core and shell structure) was found responsible for the high accumulation of nanospheres (rigid matrix system). Additionally, on the integration of all three types of nanoparticles into the hydrogel, the follicular penetration was found to be significantly declined in comparison to their liquid dispersion. The reduction in follicular penetration was due to the higher viscosity of the hydrogel. For investigating the extent of nonfollicular skin permeation (taking the negligible follicular pathway), in vitro Franz diffusion cell experiments were performed with excised skin of a human. Findings ascertained that incorporation of pure CP into hydrogel significantly reduced the cumulative amount of drug permeated (about 50%) compared to free CP in solution. It is clear that the incorporation of free CP into a hydrogel decreases the cumulative amount of CP permeated into the receptor compartment, hence the rate of permeation is about 50% with respect to free CP in solution (*p* < 0.05). A similar trend was observed for fabricated nanocarriers, whereas no significant difference was obtained between nanospheres, nanocapsules, and lipid-core nanocapsules. In this investigation, the authors presented a potential way for effective dermal delivery of CP for the management of inflammatory skin disorders [31].

### 4.7. Lecithin/Chitosan Nanoparticles

CP-loaded lecithin/chitosan nanoparticles were fabricated by Şenyiğit et al. to examine the transport of this drug (in vitro) through skin strata. The fabricated nanoparticles were characterized for zeta potential, particle size, PdI, morphology, and encapsulation efficacy. Furthermore, the nanoparticles were embedded in chitosan gel to assess the topical benefits of this nanocarrier system. Accumulation and permeation experiments of the prepared formulations were carried out using pig ear skin. The findings of this study showed epidermal accumulation of CP, with little permeation through the skin. These results indicated the minimization of side effects while improving epidermal targeting [6].

In 2017 the same research group fabricated lecithin/chitosan nanoparticles employing the self-assembling of compounds and evaluated their particle size (around 250 nm), PdI (below 0.2) and surface charge (positive). The suspension of nanoparticles was further embedded into chitosan gel for obtaining a final concentration of 0.005% CP. The anti-inflammatory potential of fabricated nanoparticles was examined employing a carrageenan-induced hind paw edema test and compared with sodium deoxycholate gel (CP 0.05% *w*/*w*) and commercial CP cream (0.05% *w*/*w*). The histopathological evaluations and transepidermal water loss study revealed a significant edema reduction in comparison to other formulations (tested). The obtained findings suggest lecithin/chitosan nanoparticles embedded chitosan gel as a promising drug delivery cargo, owing to improvement in the risk-benefit ratio for commercial cream and sodium deoxycholate gel [34].

### 4.8. Miscellaneous Cargos

In 2018 Colley and the research group fabricated mucoadhesive patches of electrospun polymer and evaluated them for their physical characteristics (film thickness, mass uniformity, swelling index, and surface morphology) and conducted cytotoxicity studies against immortalized oral keratinocytes FNB6-TERT. CP was released from the patches in a sustained pattern in both ex vivo porcine mucosa and tissue-engineered oral mucosa. Furthermore, fabricated CP-loaded patches were examined for residence time and release in animal models and were confirmed to have extended adhesion and release at therapeutic doses and times. These findings showed that fabricated mucoadhesive patches have adhered to mucosal membrane devoid of tissue damage. The fabricated patches paved the way for the management of oral conditions such as recurrent aphthous stomatitis and oral lichen planus [56].

Esfahani and his research group proposed CP-loaded chitosan patches prepared by electrophoretic deposition. Optimized chitosan patches showed 200–360% water uptake, indicating its promising swelling behavior. Moreover, the prepared patches possess flexible nature in the wet state (mechanical properties; Elastic Modulus (E) = 0.6 MPa and stress at break (σ_br_) = 0.55 MPa). Drug loading and in vitro release profiles of fabricated patches were also analyzed by ultraviolet-visible (UV-Vis) spectrophotometry. Burst release (80%) was observed in the first 2 h, which fulfilled the requirements for oral targeting. The results advocated the promising nature of CP-loaded chitosan patches for oral mucosal diseases [35].

Nanoemulgel has been explored in topical and transdermal delivery of drugs [57,58]. To enhance the therapeutic efficacy, permeation, and retention of CP in sebaceous glands, Dadwal and coworkers fabricated CP-loaded squarticles (nanoemulgels) employing the homogenization method. The fabricated nanoformulation was examined for particle size, zeta potential, viscosity, spreadability, thermal analysis, and morphology studies. In vitro release, in vitro permeation, in vivo experiments, and stability studies were also performed. The optimized nanoemulsion showed higher drug release (~84.24 ± 1.35%), whereas nanoemulgels and marketed gel displayed release of 66.83 ± 2.05% and 57.67 ± 1.63%, respectively, after 24 h. The drug retention with CP-loaded nanoemulgel was found at 63 ± 1.28%, which was more than the marketed formulation (23.12 ± 0.54%). To investigate the bio-pharmacokinetics, skin irritation, and antipsoriatic potential of CP, in vivo experiments were carried out employing Wistar rats. The inflammation was induced by UVB exposure on the rat skin. The severity scores and histopathology revealed the enhanced antipsoriatic potential of CP-loaded squarticles gel. The analysis of blood samples using HPLC indicated the slightest penetration of CP in the bloodstream and high drug deposition of nanoemulgel. These findings may be owing to the development of a CP reservoir in pilosebaceous glands owing to nanoemulsion deposition. The outcomes from irritation studies showed the safe nature of CP-loaded squarticles gel for topical application. Based on the findings from experiments, the squarticles-based gel presented a novel dosage form for the treatment of plaque psoriasis, owing to its enhanced dermal retention in skin strata [36].

For quantifying CP in follicular structures and epidermal layers, Angelo and coworkers developed the HPLC-UV method using a mixture of methanol, water, and acetonitrile (50:35:15 *v*/*v*) as a mobile phase. The optimized conditions resulted in CP elution at 10.1 min (run time: 12 min) from skin matrix obstructions. It may prove beneficial in vitro skin permeation studies [59]. Furthermore, for the management of inflammation-based skin, scalp, and hair diseases, Angelo and the research group fabricated NLCs using stearic acid, oleic acid, lecithin, and sodium taurodeoxycholate. The prepared NLCs were characterized based on particle size (180 nm with less than 0.2 PdI), thermal analysis, morphology studies, and encapsulation efficiency (80.83 ± 2.28%). The in vitro release studies displayed sustained release of the drug (50% on the 3rd day). The focus of this research was the evaluation of hair follicle uptake (employing a confocal laser scanning microscope) of prepared nanoparticles and marketed cream. Additionally, an investigation of the effect of physical stimuli (mechanical massage, ultrasound (with and without vibration), and infrared radiations (with and without metallic nanoparticles)) on follicular targeting efficacy was conducted. The confocal images ascertained enhanced epidermal follicular targeting of CP NLCs. All examined stimuli showed improved hair follicle targeting than the passive application of NLCs. Hence, NLCs presented a good choice for the delivery of CP to hair follicles [8].

### 4.9. Other Novel Formulations Reported

#### 4.9.1. Poly (d, l-lactic-co-glycolic Acid) Microspheres

In 2011, to provide prolonged-release, minimize systemic absorption, and overcome adverse effects of CP, Badilli and his research group formulated CP encapsulated poly (d, l-lactic-co-glycolic acid) (PLGA) microspheres employing an oil/water emulsion solvent evaporation method. The formulation was characterized for particle size, topography, thermal stability, and crystalline/amorphous nature. The emulgels of CP and CP-loaded microspheres were formulated and subsequently characterized and assessed for in vitro drug release. The entrapment of CP in microparticles potentially sustained its release from the emulgel delivery system, ascertaining the expectation of minimizing drug-associated side effects [37].

#### 4.9.2. Microemulsions

Microemulsions are optically isotropic, monophasic, transparent, and thermodynamically stable colloidal dispersions comprised of water, oil, co-surfactant, and surfactant with droplet sizes up to 100 nm size range. The key differences between microemulsions and nanoemulsions are based on their transparency and thermodynamic stability. Though a microemulsion has a smaller droplet size compared to a nanoemulsion both terms are still being used. Since microemulsions were first to be formulated and as such had a small droplet size, they were given the prefix micro. Nanoemulsions were later incorporated into nanometer-sized systems. To not be misled, they remained in that classification. Microemulsions are prepared via low-energy emulsification techniques [44]. Patel and his research team developed a CP encapsulated microemulsion gel for the management of vitiligo. The microformulation was optimized via a D-optional mixture experimental design by varying amounts of oil, water and surfactant, and cosurfactant mixture. The optimized microemulsion was composed of 3% oil, 50% water and 45% surfactant and cosurfactant mixture, having a globule size of 18.26 nm and CP solubility of 36.42 mg/mL. The prepared microemulsion was integrated into Carbopol^®^ 934P gel for topical application. An ex vivo permeation experiment was conducted using male Albino Wistar rat skin. Microemulsion-based gel (CP permeated-28.43 ± 0.67 μg cm^−2^) was found to possess higher retention of CP in skin layers than marketed formulations (CP permeated-37.73 ± 0.77 μg cm^−2^). The irritation studies revealed the safer nature of microemulsion-based gel with respect to microemulsion and marketed formulation. All the findings in this study advocated CP-loaded microemulsion gel as a promising dosage form for the management of vitiligo [16].

In 2014, the same research group extended their work to further validate CP microemulsion-based gel for vitiligo. The prepared formulation was characterized for gel strength, viscosity, rheology, and occlusive studies. The in vitro release studies (carried out using cellulose nitrate membrane) from the fabricated formulation exhibited higher release, enhanced accumulation, and penetration of CP in the skin when compared to the cream formulation. In vivo visualization of dermal uptake via laser scanning microscopy ascertained epidermal and skin targeting of CP. Dermatopharmacokinetic evaluation carried out in this study further revealed improvement in CP deposition in skin strata. The pilot clinical study (single-blind randomized) of microemulsion gel was evaluated by the vitiligo area score index technique. The findings from this clinical study ascertained the faster repigmentation potential of microemulsion-based gel in comparison to the control group. Hence, it was stated that CP-loaded microemulsion gel improved skin localization demonstrating good therapeutic potential in vitiligo patients [38].

For management of scalp psoriasis, Langasco and coworkers formulated CP-loaded water in oil (*w*/*o*) microemulsions. Pseudo-ternary phase diagrams were structured employing a combination of biodegradable and biocompatible excipients. In this study, Newtonian flow properties along with low viscosity and high thermodynamic stability demonstrated the suitability of microemulsions. With a higher concentration of the drug, significant changes in stratum corneum and epidermis of skin after treatment with microemulsions were observed. Ex vivo permeation analysis exhibited an improved targeted effect, which may be due to desired drug retention in upper skin annexes. Hence, the authors concluded that enhancement in CP efficacy was due to a combination of natural excipients employed for microemulsion formulation in this study [39].

#### 4.9.3. Microsponges

Devi and her research group formulated Eudragit^®^ RS 100 microsponges to minimize challenges associated with CP and to improve its delivery characteristics. The microsponges were formulated via quasi-emulsion solvent diffusion technique using Eudragit^®^ RS 100, polyvinyl alcohol, and dichloromethane. Based on maximum encapsulation efficiency, highest production yield, and sustained release, the microsponge’s optimum drug-polymer ratio was selected. The chosen formulation was found to possess an appropriate particle size range. The morphological analysis, thermogravimetry analysis, and photostability studies revealed spherical shape, thermal stability, and photostability of the prepared CP microsponges. Release kinetic analysis was performed for all the prepared batches. In vitro release from selected CP microsponges was found to follow zero-order kinetics. Further, the selected microsponge formulation was incorporated into Carbopol^®^ gel and evaluated for spreadability and viscosity. In vitro release of fabricated gel was found to follow zero-order kinetics, ascertaining that drug release was not dependent on drug concentration. In vivo antipsoriatic activity carried out using a mouse tail model revealed the remarkable improvement in pharmacological activity of CP microsponge gel. Hence, overall findings demonstrated the potential nature of CP-loaded microsponge gel with minimum side effects [4].

## 5. Cell Line Studies of Novel Clobetasol Propionate Formulations

Cell lines and cell cultures play a major role in exploring pathophysiological, physiological, and differentiation processes of particular cells for different bioactives, synthetic drugs, formulations, and marketed products. In vitro studies including these, are good alternatives and provide supplementary data for various activities. These are subjected to examine stepwise changes in the genetic makeup, biology, and structure of the cell in controlled conditions. Herein, the studies related to the efficacy of CP in various novel carriers through cell lines have been summarized (Table 2).

The ex vivo efficacy studies were carried out by Kaur et al. on HaCaT (spontaneously immortalized human keratinocytes) cell lines to perform MTT assay. The dye-loaded nanoemulsion formulations (equivalent to 1 μg/mL of free Coumarin-6) and free Coumarin-6 were used for cellular uptake studies and results were examined via confocal laser microscopy. The developed nanoemulsions presented higher cell growth inhibition in the HaCaT cell line. Enhanced drug penetration in stratum corneum and viable layers was noted which may be owing to the controlled release of CP from the nanoemulsions [23].

In the same year, in vitro cytocompatibility of CP-loaded chitin nanogel was evaluated on the L929 cell line, and the cytotoxicity of this system was examined using two cell lines i.e., HaCaT and THP1. Cellular uptake assay was conducted using HaCaT cell lines, while COX and LOX activities were examined employing THP-1 cell lines. The cytocompatibility assay employing murine dermal fibroblasts (L929 cell line) demonstrated 70% cell viability at 0.3504 mg/mL concentration with CP-loaded chitin nanogel, after 24 h of incubation, while clobetasol solution presented 65% of cell viability under the same conditions. After 48 h of the incubation period, CP nanogel showed 65% cell viability and CP solution 60% cell viability, confirming the cytocompatibility of chitin nanogels. However, the control chitin nanogel was not found to induce any remarkable cytotoxicity in comparison to the media treated cell line. A cytotoxicity study of CP-loaded chitin nanogel on HaCaT cell lines demonstrated dose-dependent toxicity with 50% cell death at a concentration of 0.35 mg/mL (incubated for 24 h) and 60% cell death (incubated for 48 h) at the same concentration. The cytotoxicity was due to CP release from the nanogel, which acted on steroid receptors of the cells. The cell cycle arrest (at S phase of DNA synthesis) by CP resulted in its antiproliferative effect, which is useful for psoriasis management since the proliferation of keratinocytes represents a hallmark of psoriasis. The fluorescent microscopy of HaCaT cells revealed particle internalization (chitin nanogel samples) in the nucleus and cytoplasm of the cells and regulated the anti-inflammatory protein expression in the nucleus and proinflammatory proteins in the cytoplasm [60]. In vitro measurements of COX and LOX expression in THP-1 cell lines demonstrated significant inhibitions by both CP chitin nanogel and control drug solution. CP-loaded chitin nanogel (at 0.35 mg/mL) presented a 78% and 63% reduction in the activity of LOX and COX, respectively without a significant difference in comparison to the control drug solution. The IC_50_ value of CP-loaded chitin nanogel in the COX inhibition and LOX inhibition was found to be 0.0904 mg/mL and 0.046 mg/mL, whereas the IC_50_ value of CP solution was observed at 0.097 mg/mL and 0.045 mg/mL for COX and LOX inhibition, respectively [7].

Recently, studies employing human HaCaT cell lines were carried out to investigate the cell viability of CP-loaded NLCs and free CP in hydroalcoholic solutions. Undiluted CP-loaded NLCs (0.05% of CP) were found nontoxic to cells (113.27 ± 13.97% viability), revealing the tolerance of NLCs at the cellular level as well as confirming the use of novel cargos for repeated long-term applications [8].

## 6. In Vivo Studies of Novel Clobetasol Propionate Formulations

The pharmacokinetic and pharmacodynamic studies of the biologically active molecule are recognized as potential tools for evaluating novel cargos. The efficacy of such molecules can be improved via employing novel delivery cargos, thereby promoting their marketing scope for pharmaceutical industries and boosting treatment strategies. The biodistribution behavior is vital for understanding the therapeutic and toxicity potential of novel cargos. The biodistribution pattern of the drug can be typically quantified based on pharmacokinetic analysis. Pharmacokinetics is often defined simply as “what the body does to the drug” and is typically described using four critical processes: absorption (A), distribution (D), metabolism (M), elimination (E), ADME. The interactions between the drug molecule (or drug delivery system) and the body control the relative rates and efficiencies of each of these processes and body compartments involved. Pharmacokinetics provide the data regarding maximum concentration (C_max_), half-life (t_1/2_), clearance (Cl), area under the curve (AUC), and mean residence time (MRT). Biodistribution means the reversible transfer of chemicals/compounds from one location to another within the body [61]. Hence, the pharmacokinetics, biodistribution, and CP-loaded therapeutic efficacy of various CP novel carriers have been explored in vivo, with the view to understanding the significance of these cargos in the management of various skin disorders. Such studies have been compiled and presented in Table 3.

Recently, the antipsoriatic potential of CP-loaded microsponge gels was examined using a mouse tail model employing albino Swiss mice. After 14 days of the treatment period, the animals were euthanized, and mice tails were subjected to histopathology analysis. Percentage of drug activity, percent orthokeratosis, and change in epidermal thickness were calculated from a histopathology evaluation. In vivo findings based on a decrease in epidermal thickness, increased orthokeratosis, and enhanced drug activity revealed a significant improvement in the efficacy of CP-loaded microsponge gel in comparison to CP gel at the same concentration (0.05% *w*/*v*). Microreservoir formation in the skin strata was proposed as the major reason for such enhanced activity [4]. Kumar and his research group recently assessed the antipsoriatic potential of the CP nanosponge-based hydrogels similarly using the mouse tail model [14].

## 7. Stability Concerns of Clobetasol Propionate

The scientific and market success of any drug product is based on in-depth knowledge of the drug discovery and development process [62]. A stability experiment is one of the critical evaluation steps for quality assurance, safety, and efficacy of the drug throughout its shelf life. It is defined as the ability of a pharmaceutical formulation in a given container or closure to lie in the range of its physical, chemical, protective, microbiological, informational, and toxicological specifications [63,64].

The ICH (International Council for Harmonisation) guidelines provide recommendations for the development of a stability-indicating process. These ICH guidelines stipulate stress testing to analyze the intrinsic stability of the drug and using the collected data to support establishing degradation patterns during the development of a dosage form [65,66]. Since gathering specific knowledge about the stability of CP is crucial for its efficacy, the current section discusses the stability issues associated with this moiety.

In 2013 Ali and the research group investigated the stability of CP-loaded nanoemulsions per ICH guidelines for three months. The shelf life of a prepared CP-loaded nanoemulsion was also examined through accelerated stability testing (40 °C ± 2 °C and 75% ± 5% relative humidity). An intermediate stability study was also performed at 30 °C ± 2 °C temperature and 65% ± 5% relative humidity. The shelf life of the optimized CP-loaded nanoemulsion was found to be 2.18 years at room temperature. The results ascertained the better chemical and physical stability of CP in nanoemulsion dosage form [67].

As per literature reports, CP is found to be stable under normal storage conditions. Formation of toxic gases is possible on heating from CP or in case of fire. Hence, it should be protected from moisture and heat. It is also found incompatible with strong oxidizing agents [67]. Further, this compound is quite unstable and susceptible to photodecomposition in the presence of light. Different research groups have studied the stability behavior of CP-loaded novel cargos under variable conditions.

In research carried out by Fontana et al. the photostability of CP-loaded nanoparticles (nanocapsules, nanospheres, and nanoemulsions) was assessed through UVA radiation exposure. All examined parameters such as size, PdI, zeta potential, and encapsulation efficiency were found to be the same up to 9 months at room temperature for CP nanocapsules. However, CP nanospheres and nanoemulsions presented an increase in their average particle size and PdI during the storage period (after 3 and 6 months, respectively). Photostability of the drug under UVA radiation was found augmented on its entrapment into nanoparticles (nanocapsules > nanoemulsions > nanospheres) [55].

In another study, Devi and her research team evaluated CP-loaded microsponge gel and CP gel for photodegradation (1 h). CP was absorbed in the UV region (presenting a peak near 237 nm), whose strength decreased on UVA exposure. The decline in CP strength observed in this study confirmed its photolysis. The findings from % CP content of CPMS and CP gel revealed the protective nature of the microsponge delivery system against UVA exposure [4]. In the following year, Kumar and his research group assessed the photostability profile of the CP-loaded nanosponges for 1 h [14].

While designing, developing, and optimizing a novel formulation, it is necessary to address several issues, particularly those related to stability. Hence, the researchers have conducted stability studies of formulations after encapsulating CP to evaluate its stability in respective delivery systems. In 2020 Yamamoto and the research team compared the pharmaceutical properties of the CP cream formulations, considering the stability of the mixture with moisturizer [68]. A brief account of such studies has been presented in Table 4.

A breakthrough in the study was demonstrated by Fauzee and Walker in an attempt to assess the degradation pattern of CP in various mediums (cream formulations, propylene glycol, and methanol) and under alkali, light, acid, neutral hydrolysis, oxidation, and heat conditions. CP degraded at 80 °C and >80 °C under oxidative, light, and basic conditions when dissolved in methanolic solutions. This moiety has a 1,4-diene-3-keto structure on ring A, which quickly undergoes deterioration in light. This RP-HPLC method is accurate, simple, linear, and precise for the separation and quantification of CP in methanol, cream formulations, and propylene glycol [65].

## 8. Safety, Tolerability, and Toxicity Concerns of Clobetasol Propionate

The commonly reported local adverse reactions associated with topical corticosteroid application include burning sensation, irritation, stinging, pruritus, erythema, folliculitis, skin chapping and cracks, numbness of the fingers, skin atrophy, and telangiectasia. These formulations have also been found responsible for hypertrichosis, dryness, secondary infection, acneiform lesions, striae, and miliaria [1,70,71]. However, systemic absorption of topical corticosteroids has been associated with reversible adrenal suppression (at relatively low doses < 50 g/week) [72,73]. Children are particularly vulnerable to corticosteroid-triggered hypothalamic-pituitary-adrenal axis suppression owing to a high ratio of body surface area to body mass. Cushing’s Syndrome and intracranial hypertension reports in pediatric patients on treatment with topical corticosteroids were found [74,75]. At relatively low doses, teratogenicity in laboratory animals was observed after systemic administration. Since there are no well-controlled studies regarding the teratogenic potential of CP in pregnant women, topical application of this drug should only be recommended after considering the risk-benefit ratio [10].

As discussed earlier, the application of corticosteroids has a considerable constructive impact on the management of inflammatory skin disorders. The ultrapotent agents such as CP present an effective option for the treatment of psoriasis[3,76]. Since 1973 it has been employed for the management of corticosteroid-sensitive skin conditions, and several preclinical trials for tolerability and safety of its novel formulations are summarized here.

Irritation is commonly associated with the side effect of corticosteroids, particularly CP, which may limit its utility. Hence, some research groups have explored this side effect in their fabricated formulations. In 2013 Patel and colleagues studied the irritation potential of CP-loaded microemulsions and their gels using Driaze primary skin irritation assay. In this study microemulsion gel showed remarkable improvement over a marketed formulation, in terms of erythema, edema, and irritation scores. This was due to a change in microemulsion properties on the addition of Carbopol^®^. Furthermore, the three-dimensional network structure and augmented viscosity of Carbopol^®^ lower the probability of direct contact of a microemulsion-loaded drug to applied skin areas [16]. In another study, Alam et al. selected the materials, which were recognized as a safe (GRAS) category for fabrication of nanoemulsions. Skin irritation potential was assessed for 14 days using Wistar rats to ascertain the safety of the nanoemulsion. The average skin irritation score estimated for placebo nanoemulsion gel and drug-loaded nanoemulsion gel was found to be 0.83 ± 0.75 and 1.66 ± 0.81, respectively. The outcomes of this study ascertained the safe nature of nanoemulsion gel for human use [21,22].

In the following year, Kaur and her research group checked nanoemulsion for skin irritation using dorsal skin (hairless) of rats. Minor irritation was noted in the case of nanoemulsion gel-treated animals. In this formulation, calcipotriol has an affinity to cause skin irritation, but a larger quantity of CP penetrating the skin could cover the irritation that occurred due to calcipotriol. Another cause of minimal irritation could be controlled exposure and sustained release of drugs to the skin. In addition, nanoemulsion gel having active moieties in entrapped form, presents minimum contact with the skin [23]. In the same year, Panonnummal et al. examined the skin irritation potential of CP chitin nanogel applied in repeated doses for 2 weeks and compared it with the corresponding marketed product. CP chitin nanogel did not induce any perceptible variations on mice skin, whereas slight depigmentation and marked wrinkles were observed in the case of the commercial drug products [7]. Recently, in another formulation strategy, Dadwal et al. reduced skin irritation of CP by loading it in squarticle-based gels [36].

## 9. Conclusions and Future Prospectus

CP has emerged as an extremely promising anti-inflammatory molecule due to its vast therapeutic prospects for many decades in medicinal practice worldwide. However, side effects including skin atrophy, allergic contact dermatitis, steroidal acne, Cushing’s syndrome, hypopigmentation, and skin irritation associated with this drug hamper its utility in clinical scenarios to treat skin disorders. Furthermore, its poor aqueous solubility and light sensitivity pose a challenge for formulation scientists. Extensive pharmaceutical research on this moiety has been conducted in the past to improve its water solubility, stability, bioavailability, and minimize CP-associated side effects. In this context researchers have paid particular attention and explored a variety of novel delivery cargos for CP, such as microspheres, microsponges, microemulsions, nanosponges, nanocapsules, nanospheres, polymeric nanoparticles, nanoemulsions, SLNs, NLCs, and nanogels. Such novel formulations can overcome the above-mentioned challenges by their efficient skin targeting.

Although CP-loaded novel cargos displayed superior safety patterns in both humans and animals, more research should be conducted to explore the toxicity of these formulations, mainly after frequent administration of high doses. It is well known that all delivery carriers have their inherent benefits and limitations for a particular application, and it is essential to choose the most appropriate formulation for selected moiety. It was observed that the entrapment of CP into novel drug carriers successfully overcomes the various barriers of the skin. It is worth mentioning here that CP-incorporated novel cargos will also help in their dose reduction, which subsequently result in fewer side effects and better patient compliance. However, nanocarriers of this moiety are still in their infancy, and keeping in mind their potential, it will be essential to explore these carriers for ease of scale-up, ease of fabrication, cost, environmental impact, and sustainability. This will help in obtaining commercial products of CP nanoformulations in the near future.

Moreover, CP and its combination with other moieties such as calcipotriol and tacrolimus have been investigated for improvement of antipsoriatic effects and higher dermal penetration. These formulations have revealed superior therapeutic properties in comparison to free CP. These moieties can either be combined in their free form or, preferably, be fabricated in the same carrier or integrated with suitable topical vehicles (hydrogels, creams, or emulgels). Additionally, the knowledge of pathology in various skin ailments is an essential aspect for choosing the proper target and designing novel cargos for CP. The cost-benefit ratio of novel dermatological formulations and free CP in these drug products also needs to be investigated.

Clinical studies certainly draw attention to the potential of CP-based novel nanoformulations, which are lacking in the literature and must be validated. So far, the studies on CP novel formulations are mostly on psoriasis treatment, but a few have revealed that the formulations have prospective uses for the management of other chronic skin ailments including eczema, vitiligo, contact dermatitis recurrent aphthous stomatitis, and oral lichen planus. Broad human clinical trials have to be carried out to ascertain their safety, particularly after repeated and chronic use, and usefulness for the management of dermal ailments.

## Figures and Tables

**Table 1:** Novel formulations reported for clobetasol propionate since 2010.

Carrier Systems	Fabrication Methods	Evaluation	References
Nanoemulsions	Aqueous phase titration method	In vivo anticontact dermatitis, anti-inflammatory, and irritation studies using Wistar rats	[21]
Rich algal oil nanoemulsion gel	Aqueous phase titration method	In vivo skin irritation, anti-inflammatory studies, and Nucleoside triphosphate phosphohydrolase activity of lymphocytes	[22]
Nanoemulsion-loaded gels	Spontaneous emulsification method	Codelivery of CP and calcipotriol for the management of psoriasis with dermatokinetics and skin distribution	[23]
Lipid-core nanocapsules, nanoemulsions	Interfacial deposition of the polymers, spontaneous emulsification	In vivo efficacy against contact dermatitis and Nucleoside triphosphate phosphohydrolase activity of lymphocytes using female Wistar rats	[24]
Chitin nanogels	Controlled regeneration method	In vitro and in vivo antipsoriatic studies with oxidative stress markers	[7]
SLNs	Emulsification–homogenization method	Ex vivo diffusion study	[25]
Nanostructured lipid carriers	Microemulsion technique	CP accumulation in stratum corneum in porcine ear skin	[26]
Nanostructured lipid carrier gels	The hot high-pressure homogenization method	In vitro release; in vivo anti-inflammatory assay using Wistar albino rats	[27]
Nanostructured lipid carriers	Microemulsion technique	Epidermal targeting and permeation studies using porcine ear skin	[28]
Nanocapsules	Interfacial deposition of the polymers	In vitro drug release and photo stability	[29]
Nanocapsules	Interfacial deposition	In vivo induction and treatment of contact dermatitis in female Wistar rats and oxidative stress assessment in liver tissue	[30]
Nanospheres, nanocapsules, lipid-core nanocapsules	Nanoprecipitation-solvent evaporation technique	Optimization between interfollicular permeation and follicular uptake balance to minimize adverse effects	[31]
Lipid nanoparticles	Microemulsion technique	Co-delivery with tacrolimus	[32]
Hybrid nanoparticles	Monowave assisted ring-opening polymerization	In vivo antipsoriatic activity	[33]
Lecithin/chitosan nanoparticles	Ionic interaction	Evaluation of skin barrier function and damage	[34]
Chitosan patches	Electrophoretic deposition	For fast drug delivery in oral mucosa disease	[35]
Squarticles (nanoemulgels)	Homogenization method	Enhancing the better permeation, increasing skin retention	[36]
PLGA microspheres	Oil/water emulsion-solvent evaporation method	In vitro drug release studies with sustained release	[37]
Microemulsion based gels	Homogenization method	Ex vivo skin permeation on male Wistar albino rat skin and in vivo skin irritation studies on Albino rabbits	[16]
Microemulsion based gels	Homogenization method	In vivo dermatokinetics and pilot clinical studies for vitiligo treatment	[38]
Microemulsions	Homogenization method	Drug distribution through microscopy, ex vivo skin permeation studies	[39]
Eudragit microsponge gels	Quasi emulsion solvent diffusion method	Therapeutic efficacy of the drug for psoriasis	[4]
Lipid nanocarriers	Microemulsion technique	In vitro cutaneous permeation, in vivo hair follicle targeting with physical stimuli (IR, US, mechanical message)	[8]
Lipid-core nanocapsule gels	Interfacial deposition of preformed polymers	In vitro skin permeation and penetration in abdominal porcine skin	[40]
Cyclodextrin based nanosponge hydrogel	Melt method	In vivo antipsoriatic activity	[14]

**Table 2:** Cell line reports for novel formulations of clobetasol propionate.

Carrier Systems	Cell Lines	Assays	References
Nanoemulsion-loaded gels	HaCaT	Ex vivo efficacy study (MTT assay)	[23]
Chitin nanogels	L929, HaCaT, and THP1	Cyto-compatibility, Cell uptake study, COX and LOX activity	[7]
Mucoadhesive patches	Immortalized oral keratinocytes FNB6-TERT	Cytotoxicity studies	[56]
Hybrid nanoparticles	HaCaT	Cellular uptake studies, in vitro cytotoxicity assay, apoptosis assay, and Cell-cycle analysis	[33]
Nanostructured lipid carriers	HaCaT	Cell viability study	[8]
Nanosponge hydrogels	THP1	Cytocompatibility studies	[14]

**Table 3:** In vivo studies of novel cargos of clobetasol propionate.

Delivery Systems	Animals Used	Activity/Bioassay	Remarks	References
Lipid-core nanocapsules, nanoemulsions	Female Wistar rats	5% Nickle sulfate-induced dermatitis, NTPDase activity of lymphocytes	Enhanced NTPDase activity using lipid core nanocapsule-loaded hydrogels	[24]
Nanocapsule loaded hydrogels	Female Wistar rats	Nickle sulfate-induced dermatitis, biochemical assays of liver	Enhanced protective action against the oxidative damage using CP-loaded nanocapsules	[30]
Nanoemulsions	Wistar rats	Anti-inflammatory activity (Hind paw edema method)	Maximum inhibition of edema observed with prepared formulation	[21]
Nanoemulsions	Wistar rats	Skin irritation test	The formulation showed low irritation potential	[21]
Microemulsion based gels	Albino rabbits	Skin irritation test	Microemulsion-based gel found to be less irritant than marketed formulation	[16]
Microemulsion based gels	Albino Wistar rats	Dermatopharmacokinetic study	Enhanced therapeutic activity at the site of action and improvement in bioavailability	[38]
Nanoemulsions	Wistar rats of either sex	Anti-inflammatory activity (Hind paw edema method)	Hydrogel-thickened nanogel formulation has better anti-inflammatory activity than plain gel	[22]
Nanoemulsions	Wistar rats of either sex	Skin irritation test	Nanoemulsion showed more irritation potential than placebo formulation but was found safe for human use	[22]
NLCs	Male Wistar rats	Anti-inflammatory activity (paw edema method)	Decreased inflammation for a longer period was demonstrated by using NLCs	[27]
Nanoemulsion-loaded gels	Balb C mice	Antipsoriatic activity	Nanoemulsion-loaded gel displayed maximum antipsoriatic activity in comparison to plain gel and marketed formulation	[23]
Nanoemulsion-loaded gels	Balb C mice	Skin irritation test	Nanoemulsion-loaded gel showed very low irritation potential as compared to plain gel and marketed formulation	[23]
Nanogels	Balb C mice	Imiquimod induced psoriasis model	Nanogel presented better antipsoriatic activity than marketed formulation	[7]
Nanogels	Balb C mice	Skin irritation test	Nanogel was not found to induce any noticeable changes on the mice back skin	[7]
Nanoparticles	Male albino Wistar rats	Anti-inflammatory activity (carrageenan-induced hind paw edema model	Nanoparticles demonstrated significantly higher anti-inflammatory activity when compared to a sodium deoxycholate gel and commercial cream (Dermovate) containing the same drug.	[34]
Microsponge based gels	Swiss albino mice	Antipsoriatic activity (mouse tail model)	Microsponges displayed a higher efficacy than plain gel	[4]
Hybrid nanoparticles	Swiss albino mice	Antipsoriatic activity (imiquimod induced psoriasis-like inflammation)	Enhanced antipsoriatic potential	[33]
Squarticles (nanoemulgels)	Wistar rats	Ultraviolet B exposure; Skin irritation study and pharmacokinetic study	Enhanced antipsoriatic activity compared to marketed formulation, no sign of skin irritation, least penetration of the CP in the blood, and high CP deposition in pilosebaceous glands was observed	[36]
Nanosponge hydrogels	Swiss mice	Antipsoriatic activity (mouse tail model)	Enhanced antipsoriatic potential compared to plain CP gel	[14]

**Table 4:** Stability studies of the CP novel formulations.

Carrier Systems	Storage Conditions	Evaluation	References
Nanocapsules, nanospheres, and nanoemulsions	Kept in dark at room temperature (25 ± 2 °C) for 9 months	Drug content, pH, encapsulation efficiency, particle size, PdI, and zeta potential	[55]
Nanocapsules	Stored in dark at room temperature for 3 months	Particle size, PdI, and zeta potential	[29]
Lecithin/chitosan nanoparticles and their gels	25 °C and 60% RH for 3 months	Particle size, PdI, and zeta potential for nanoparticles; pH, viscosity, and drug content for nanoparticle-based gels	[6]
Nanoemulsions	40 °C ± 2 °C/75% ± 5% RH; 30 °C ± 2 °C/65% RH ± 5% RH	Accelerated stability studies; Shelf life of nanoemulsions	[67]
Microemulsions and microemulsion based gels	2–8 °C and 40 ± 2 °C/75 ± 5% RH for three months	Globule size and PdI for microemulsions, appearance for microemulsion-based gel	[16]
Tea tree oil nanoemulsion	As per ICH guidelines for 3 months	Accelerated stability studies, Phase separation, Ostwald ripening, coalescence, and creaming	[69]
Nanoemulsion gel	Centrifugation (5000 rpm) for 30 min, heating and cooling cycles, and Freeze-thaw cycles	Physical stability studies	[22]
Nanocarriers	Room temperature (25 ± 2 °C) for 3 months	Particle size, PdI, pH, and zeta potential	[31]
Nanostructured lipid gel	5 ± 1, 25 ± 2, 40 ± 2, 60 ± 2 °C, and 75 ± 5% RH for 6 months	Shelf life of the prepared formulation	[27]
NLCs	Room temperature (25 ± 2 °C) 4 °C for 7 days	Colloidal stability assessment using Turbiscan Lab apparatus for 90 min	[28]
Chitin nanogels	2–8 °C, 25 ± 5 °C and 40 °C with 65% RH for 3 months	Appearance, physical state, odor, color, and particle size	[7]
Bio-based microemulsions	Centrifugation (13,000 rpm) for 30 min; Also, at 2–8 °C and room temperature (25 ± 2 °C)	Physical stability studies	[39]
Microsponge gel	5 ± 2 °C, 25 ± 2 °C and 40 ± 2 °C for 40 days	Appearance, pH, drug content, and in vitro release pattern	[4]
NLCs	5, 25 and 40 °C for 30 days	Hydrodynamic diameter, PdI, zeta potential, pH, and entrapment efficiency	[8]
Squarticles based gel	4 ± 2, 25 ± 2 and 45 ± 2 °C for 6 months	Entrapment efficiency, PdI, particle size, and drug content at periodical intervals	[36]
Nanosponges	25 °C for 3 months	Particle size, zeta potential, PdI, and drug content	[14]

## Data Availability

The data presented in this study are contained within the article.

## Abbreviations

COX	cyclooxygenase
CP	clobetasol propionate
DUSP	dual-specificity protein phosphate
DUSP-1	dual-specificity protein phosphatase 1
GR	glucocorticoid receptors
GREs	glucocorticoid-responsive elements
Hsp90	heat-shock protein 90
ICH	International Conference on Harmonization
IL-1	interleukin 1
IκBa	inhibitory nuclear factor-κBa
LCNC	loaded lipid core nanocapsule
LOX	lipoxygenase
mRNA	messenger RNA
MTT	3-(4,5-Dimethylthiazol-2-yl)-2,5-Diphenyltetrazolium bromide
NF-κb	nuclear factor kappa-light-chain-enhancer
NLCs	nanostructured lipid carriers
NTPDase	nucleoside triphosphate phosphohydrolase
PdI	polydispersity index
PEPCK	phosphoenolpyruvate carboxykinase
PLA	poly(DL-lactide)
PLGA	poly(lactic-co-glycolic acid)
SLNs	solid lipid nanoparticles
TAT	tyrosine aminotransferase
US-FDA	United States-Food and Drug Administration
UV-Vis	ultraviolet-visible
